# Supplementary material for: Conformational coupling of the sialic acid TRAP transporter HiSiaQM with its substrate binding protein HiSiaP
Source: Nat Commun. 2024 Jan 8;15:217. doi: 10.1038/s41467-023-44327-3 (PMC10774421; doi:10.1038/s41467-023-44327-3)
Supplement: Supplementary file 1 — New Supplementary Information [file 41467_2023_44327_MOESM1_ESM.pdf]

Supplementary information for

Conformational coupling of the sialic acid TRAP transporter HiSiaQM with its substrate binding protein HiSiaP

Martin F. Peter[1,2#], Jan A. Ruland[3,#], Yeojin Kim[1,#], Philipp Hendricks[1,#], Niels Schneberger[1], Jan Peter Siebrasse[3], Gavin H. Thomas[4], Ulrich Kubitscheck[3], Gregor Hagelueken[1,*]

[1] Institute of Structural Biology, University of Bonn, Venusberg-Campus 1, 53127 Bonn, Germany

[2] Present address: Biochemistry Center, Heidelberg University, Im Neuenheimer Feld 328, 69120 Heidelberg, Germany

[3] Clausius Institute for Physical und Theoretical Chemistry, University of Bonn, Wegelerstr. 12, 53127 Bonn, Germany

[4] Department of Biology (Area 10), University of York, York YO10 5YW, United Kingdom

[#]equal contribution

*Corresponding author email: hagelueken@uni-bonn.de

Supplementary Table 1 | Details of the engineered disulfide bridges

**Construct**	**C_β distance (Å)**	**Based on model**
CLOSED #1 (HiSiaP S44C-S171C)	4.8	3B50 [6]
CLOSED #2 (HiSiaP S15C-A194C)	4.0	3B50 [6]
IFS #1 (HiSiaQM$_{\Delta Cys}$ A492C-Q539C)	5.9	7QE5 [14]
IFS #2 (HiSiaQM$_{\Delta Cys}$ M297C-M259C)	4.8	7QE5 [14]
OFS #1 (HiSiaQM$_{\Delta Cys}$ A492C-L512C)	6.1	Model of OFS[14]
OFS #2 (HiSiaQM$_{\Delta Cys}$ M297C-Y252C)	5.0	Model of OFS[14]

Supplementary Table 2 | Data collection and refinement statistics

PDB-ID	8CP7
Space group	I222
Unit Cell dimensions (Å)	
a, b, c	84.08, 89.45, 90.5
α, β, ɣ	90, 90, 90,
Resolution (Å)	42.04 - 1.9 (1.97-1.9)
Wilson B-factor (Å^2)	33.96
R_{merge}	0.02891 (0.6132)
$CC_{1/2}$	1.0 (0.721)
I/σ(I)	14.84 (1.14)
Completeness	99.02 (99.43)
Redundancy	2.0(2.0)
Unique reflections	26990(2437)
R_{work}	0.2105 (0.3441)
R_{free}	0.2589 (0.3640)
Average B factor (Å^2)	39.37
Protein	39.47
Ligands	31.99
Solvent	38.66
Non-hydrogen atoms	2635
Protein	2478
Ligands	23
solvent	134
RMS (bonds) (Å)	0.010
RMS (angles) (°)	1.01
Ramachandran (%)	
favoured	99.35
allowed	0.65
outliers	0.00
Clashscore	4.2

Values in parentheses refer to the high-resolution shell.

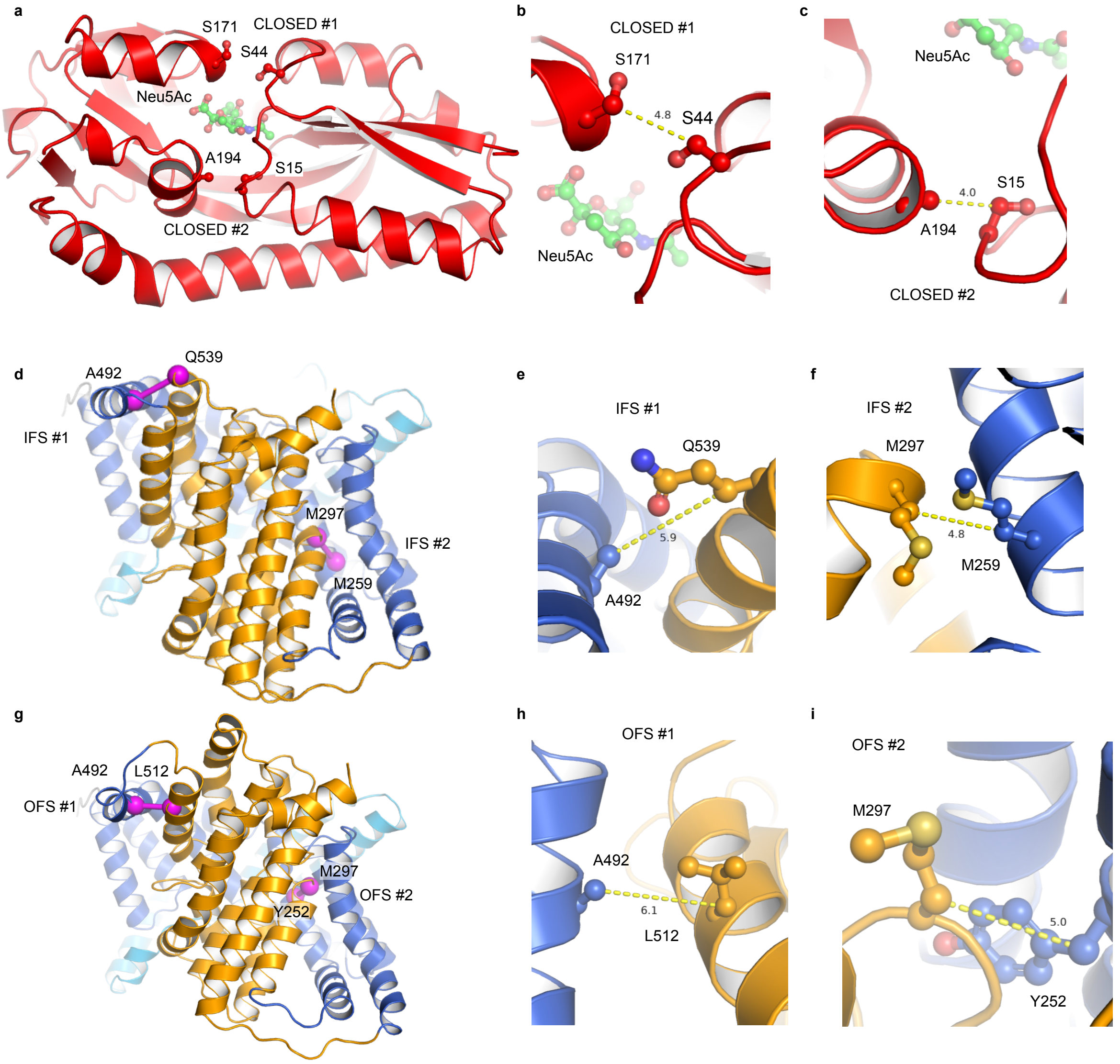

Supplementary Fig. 1 | Design of disulfide linked constructs for HiSiaP and HiSiaQM. a) Cartoon representation of HiSiaP with Neu5Ac as green sticks and the positions that were mutated to cysteines in CLOSED #1/2 shown as sticks. **b**) Close-up of the CLOSED #1 site. **c**) Close-up of the CLOSED #2 site. **d**) Structure of HiSiaQM in the IFS shown as cartoon model with the positions that were mutated to cysteines shown as magenta spheres. **e,f)** Close-up of the IFS#1/2 sites. **g**) Model of HiSiaQM in the OFS shown as cartoon model with the positions that were mutated to cysteines shown as magenta spheres. **h,i)** Close-up of the OFS#1/2 sites. IFS: inward-facing state; OFS: outward-facing state.

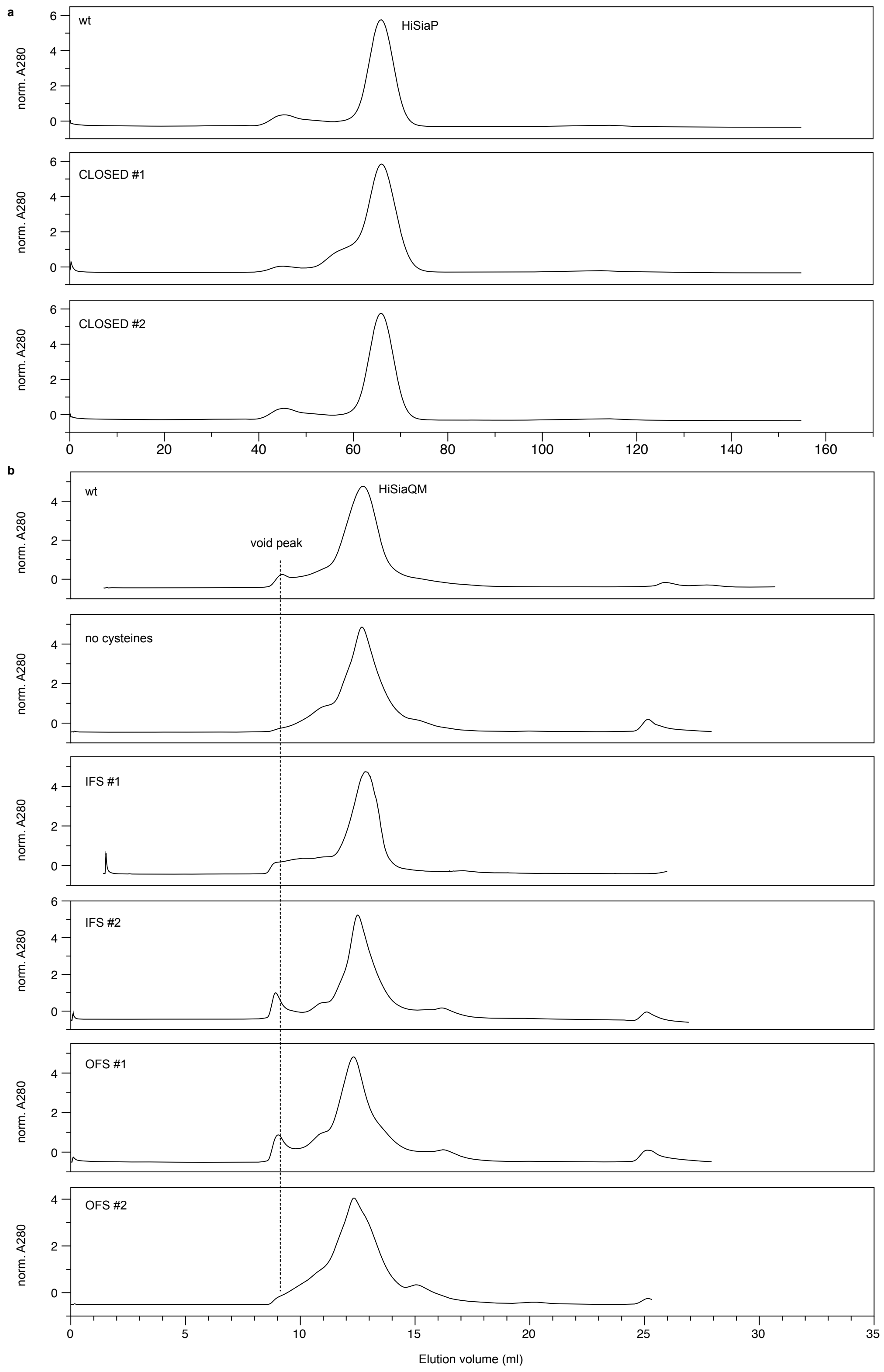

Supplementary Fig. 2 | Gelfiltration runs of the different HiSiaP and HiSiaQM constructs used in this study. a) All HiSiaP gelfiltrations were performed on a Superdex 75 16/600 column. **b)** All HiSiaQM gelfiltrations were performed on Superdex 200 10/300 columns but on different FPLC systems. Therefore, the runs were aligned on the void peak for easier comparison. FPLC: Fast protein liquid chromatography. Source data are provided as a Source Data file.

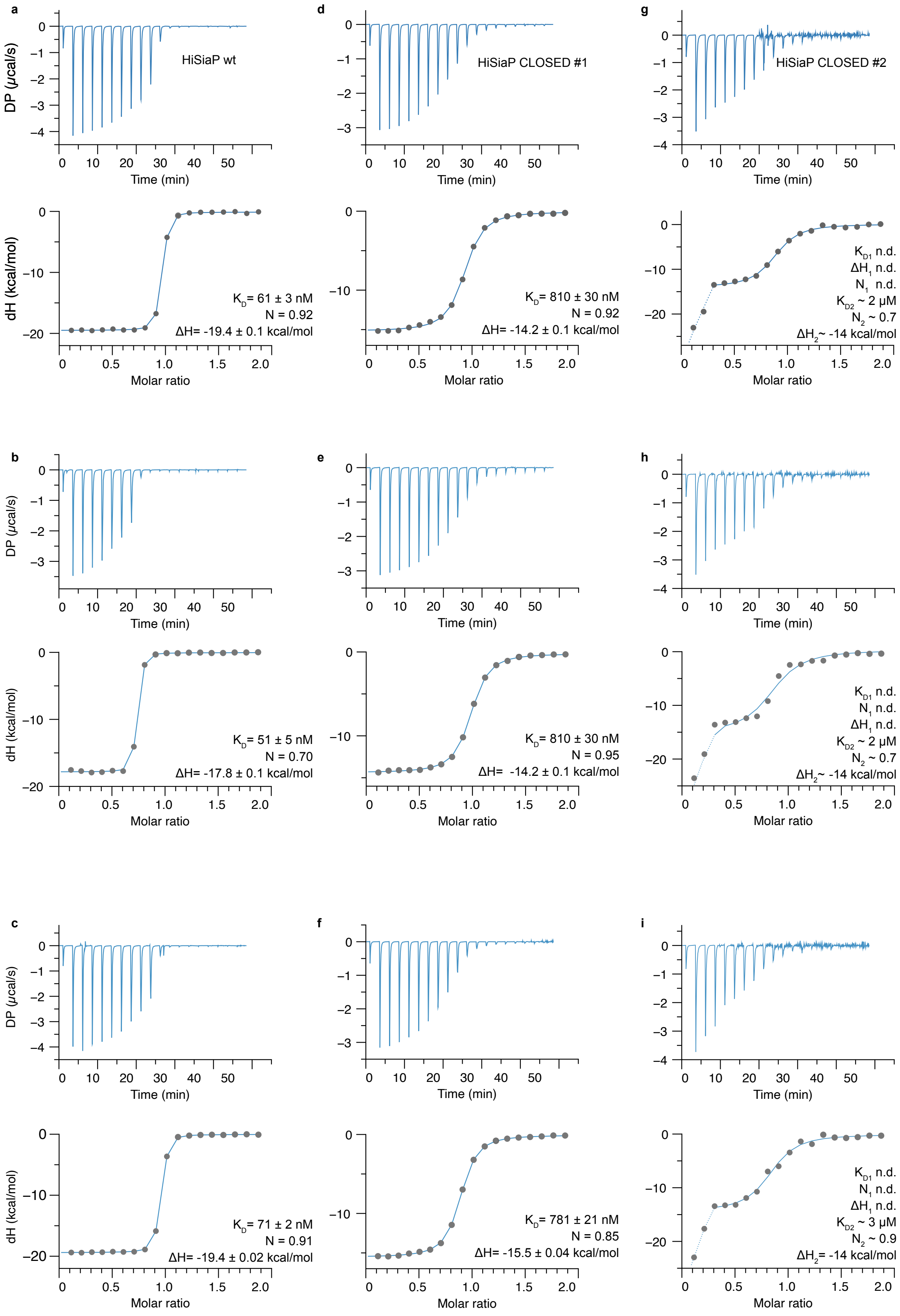

Supplementary Fig. 3 | Isothermal titration calorimetry runs for the indicated HiSiaP constructs. In each panel, the raw data are shown on the top and the integrated heat signals, as well as the thermodynamic parameters at the bottom. Source data are provided as a Source Data file.

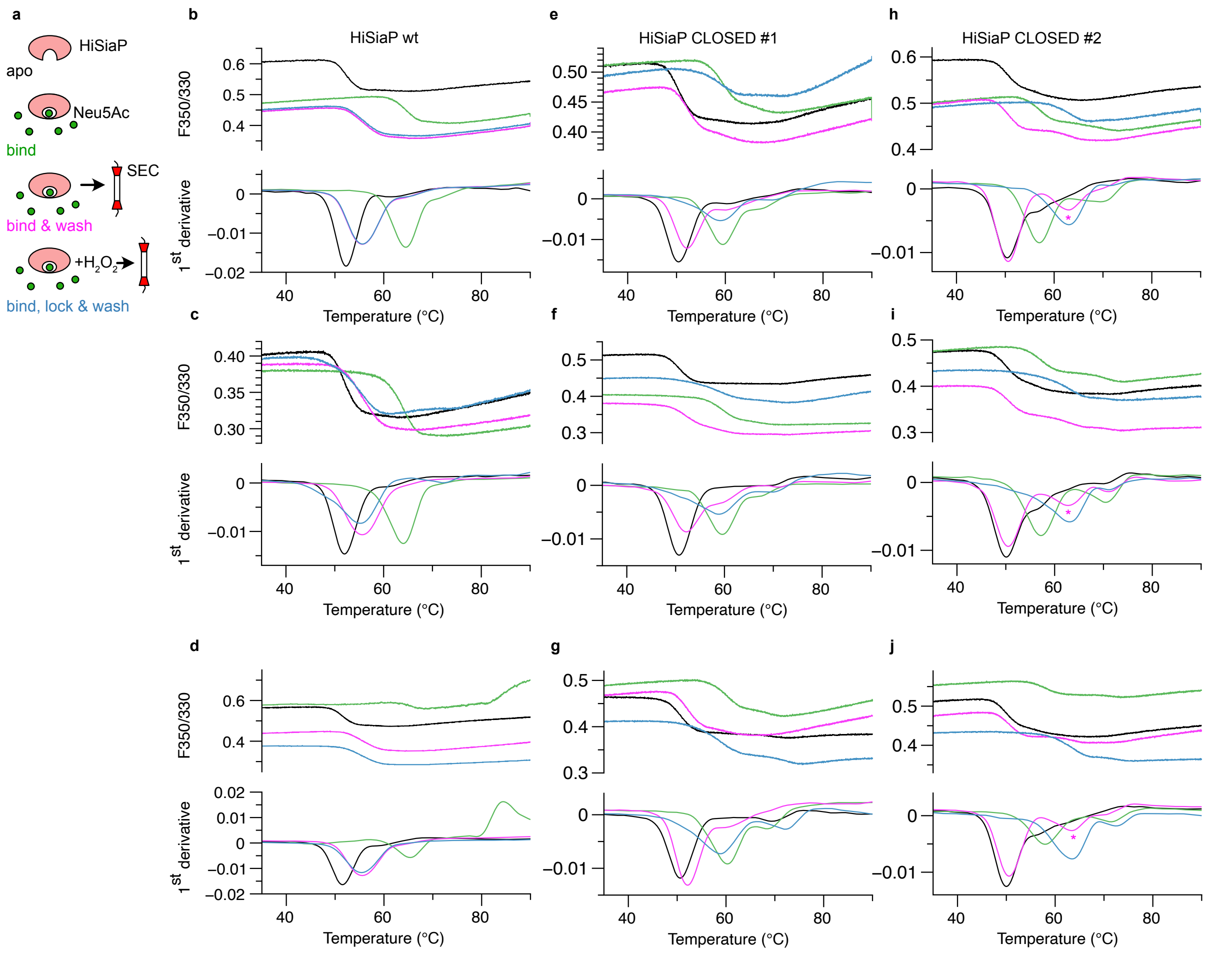

Supplementary Fig. 4 | NanoDSF Experiments on the HiSiaP constructs used in this study. a) Schematic as in Fig. 2, explaining the setup of the different experiments and the color code of the traces in panels b-j. **b)** NanoDSF runs with raw data shown on the top and the 1st derivative of each trace shown on the bottom. The color code is explained in panel a). The magenta asterisks in panels h-j) correspond to the magenta asterisk in Fig. 2b and mark the position of the distinct second peak in the 1st derivative of the "bind & wash" experiment for HiSiaP CLOSED #2. DSF: Differential scanning fluorimetry. Source data are provided as a Source Data file.

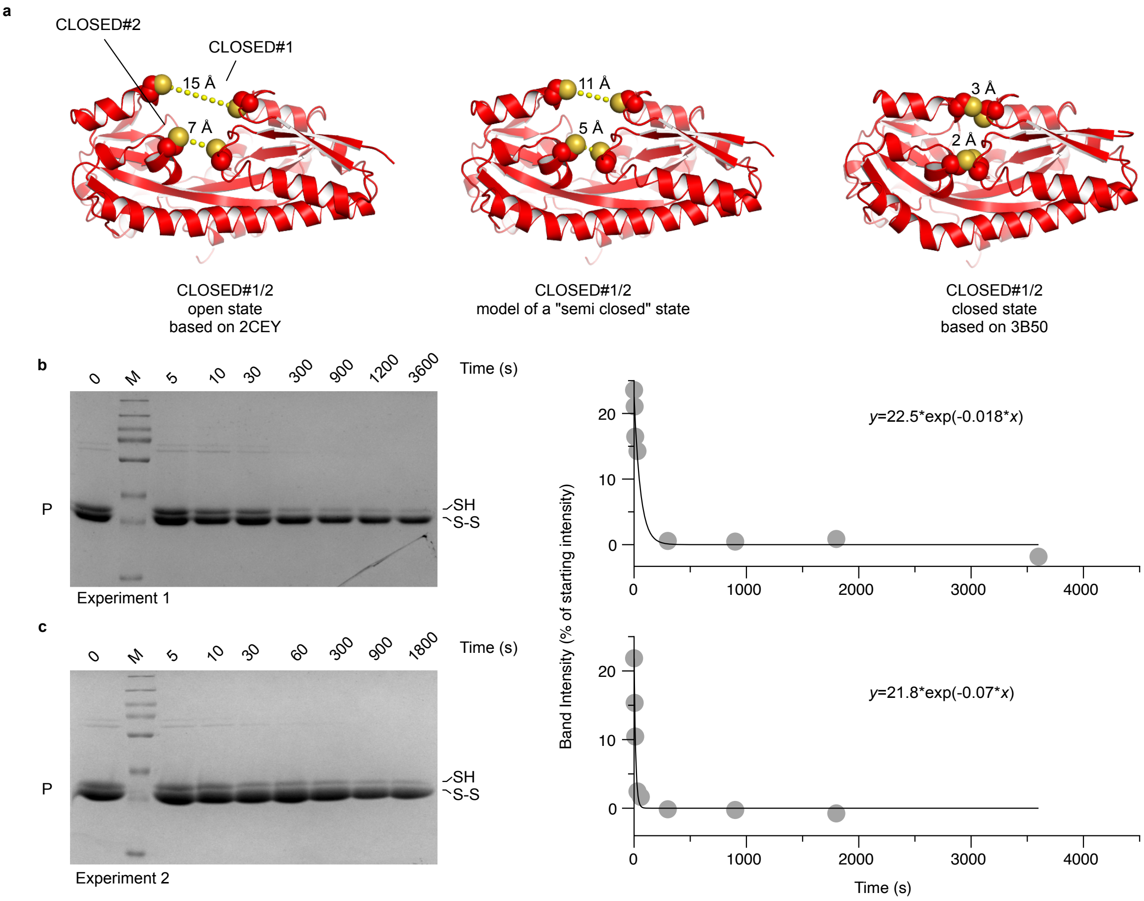

Supplementary Fig. 5 | Trapping rare closing events of apo HiSiaP. a) The cartoon models show HiSiaP in its open (left), closed (right) and a modelled semi-closed state (middle). The model was produced using Rigimol (www.pymol.org). The engineered cysteine residues are shown as spheres and the distances between the S_γ atoms are shown as dotted yellow lines. In this model, due to its position with respect to the hinge of the closing motion, the CLOSED #2 cysteines can form a disulfide bond, when the CLOSED #1 cysteines are still too far apart. **b, c)** left: Two (n = 2) independent time course experiments of apo HiSiaP CLOSED #2 forming a disulfide link in the presence of copper phenantroline. right: The intensities of the bands on the left were quantified and plotted as a percentage of the initial intensity of the upper band, i.e. of the non-crosslinked fraction of HiSiaP (grey circles). The intensities could be fitted with a mono exponential decay function. The fitting equation is given for each experiment. Uncropped version of the two gels are shown in Supplementary Fig. 10. Source data are provided as a Source Data file.

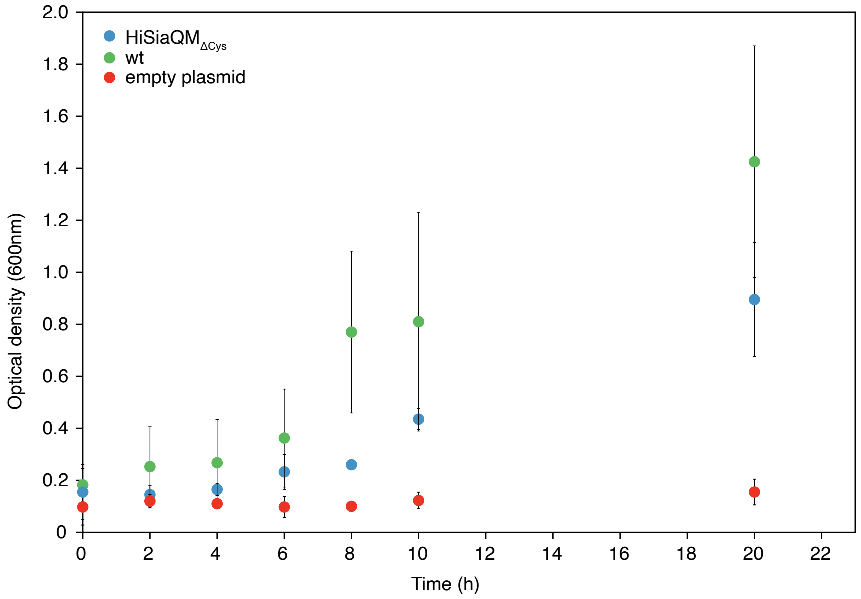

Supplementary Fig. 6 | A sialic acid uptake assay as in SEVY3-based complementation assay [14,24,25]. Briefly, *E. coli* cells without a functioning sialic acid uptake system were supplemented with either wild-type HiSiaPQM, the HiSiaPQM$_{\Delta Cys}$ mutant or an empty plasmid. Growth of these bacteria was monitored in minimal media with sialic acid as the sole source of carbon and energy. Data of n=3 independent experiments are presented as mean values (colored circles) +/- standard deviation (error bars). Source data are provided as a Source Data file.

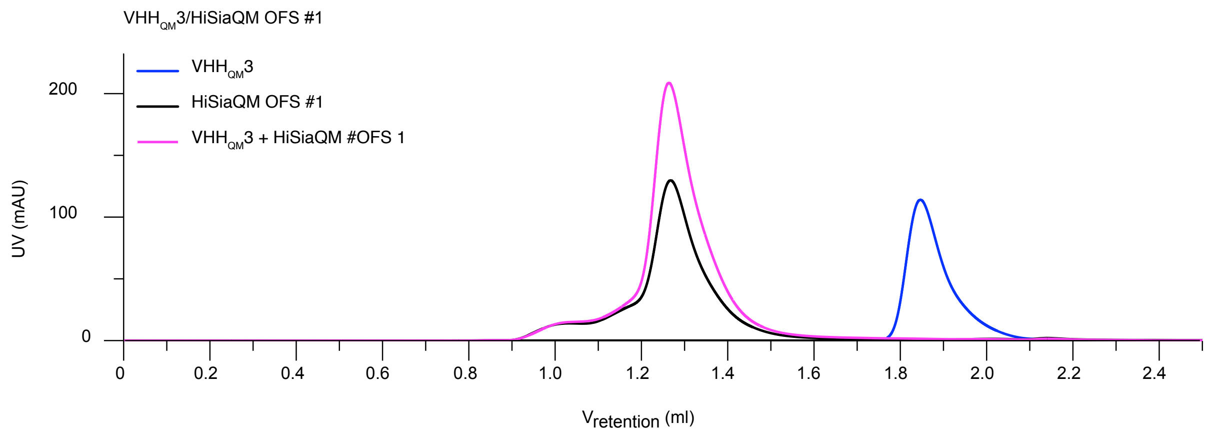

Supplementary Fig. 7 | HiSiaQM OFS#1 in detergent binds VHHQM 3. Three gel filtration experiments (Superose increase 6 3.2/300) with equal amounts of either VHH$_{QM}$ 3 (blue), HiSiaQM OFS#1 (black) or a mix of both components (magenta). The experiment shows that in DDM detergent, VHHQM 3 (blue curve) binds to HiSiaQM OFS#1. OFS: outward-facing state. Source data are provided as a Source Data file.

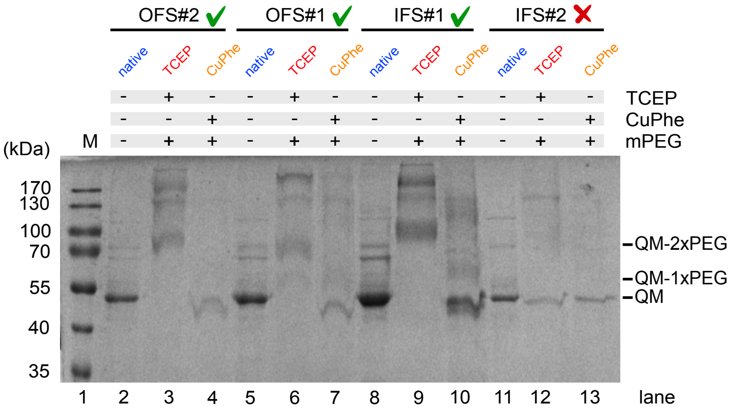

Supplementary Fig. 8 | Example of the PEGylation experiment as described in Fig. 4f. Results that met our expectations are marked with a green checkmark. Validation results that did not meet our expectations are marked with a red cross. An uncropped version of this gel is shown in Supplementary Fig. 10. PEG: polyethylene glycol.

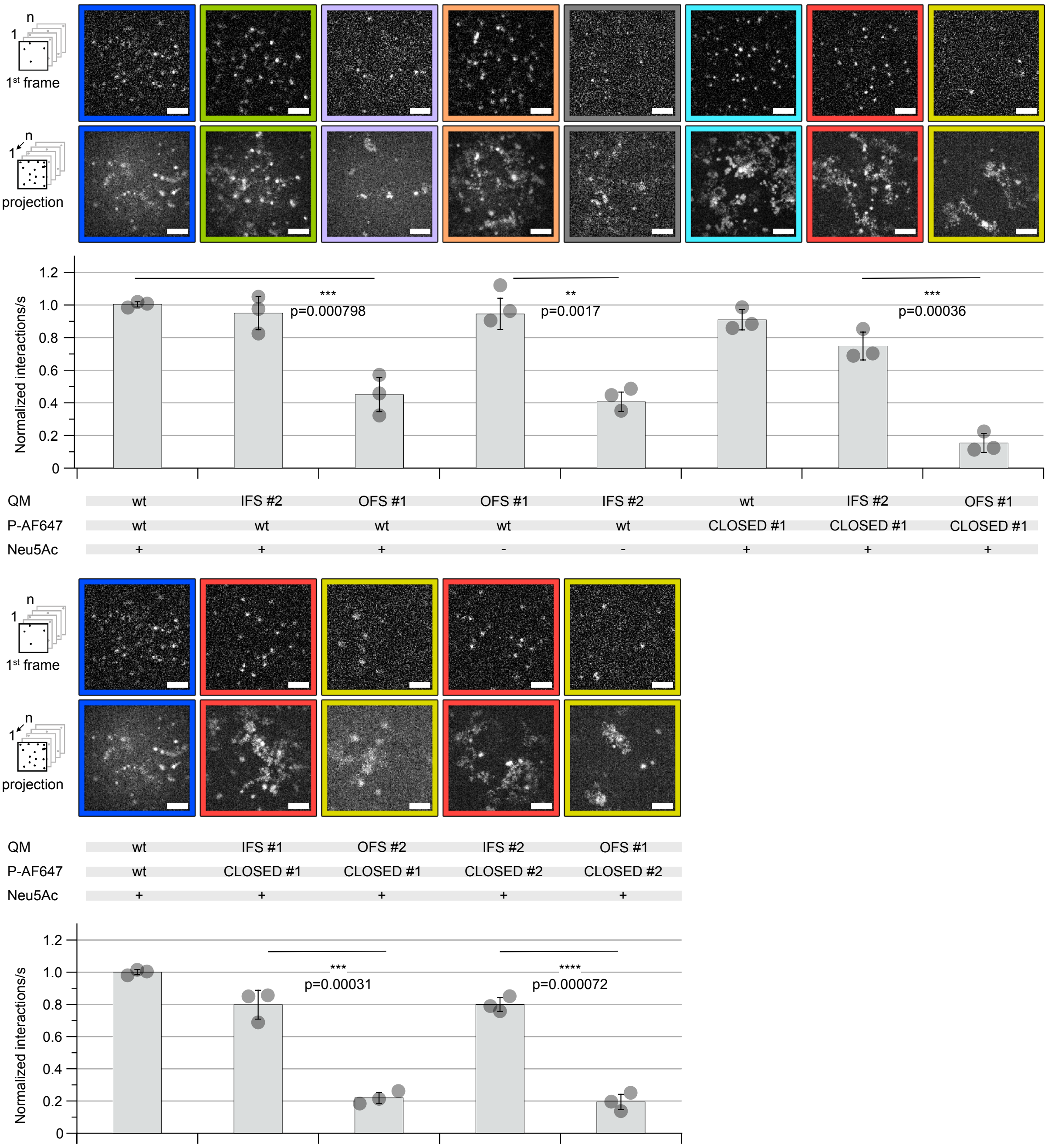

Supplementary Fig. 9 | Single molecule TIRF microscopy of trapped TRAP transporter domains. a-l) Top: first frame of an image sequence of a typical set of data and the corresponding maximum intensity projection of the respective image sequence. Bottom: Normalized interactions per second between AF647 labelled HiSiaP constructs and the indicated HiSiaQM constructs. The statistical significance of differences between selected experiments was assessed by applying a two-sided unpaired Student's t-test with a 95% confidence interval. The scale bars equal 3 µm. A movie with all conditions can be found in the supplementary information (Supplementary Movie 1). TIRF: total internal reflection. Source data are provided as a Source Data file.

Fig. 2d

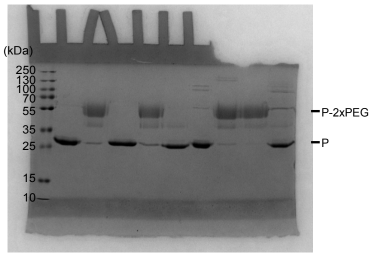

Fig. 2e

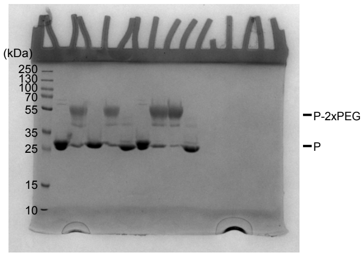

Fig. 4g

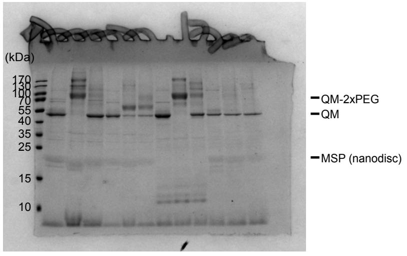

Supplementary Fig. 8

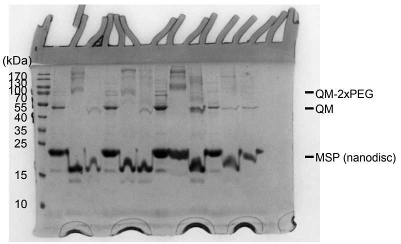

Supplementary Fig. 5b

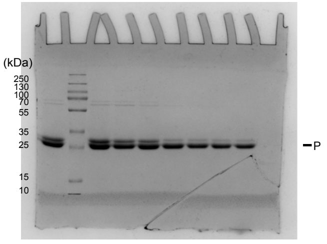

Supplementary Fig. 5c

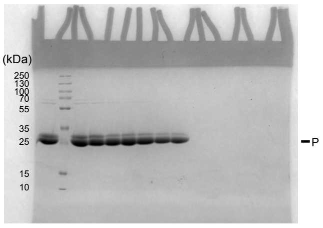

Supplementary Fig. 10 | Uncropped versions of the gels throughout the manuscript. In each panel the Figure with the cropped version of the gel is indicated.
