# Supplementary material for: Conformational coupling of the sialic acid TRAP transporter HiSiaQM with its substrate binding protein HiSiaP
Source: Nat Commun. 2024 Jan 8;15:217. doi: 10.1038/s41467-023-44327-3 (PMC10774421; doi:10.1038/s41467-023-44327-3)
Supplement: Supplementary file 3 — Description of Additional Supplementary Files [file 41467_2023_44327_MOESM3_ESM.pdf]

Title: Supplementary Movie 1:
Description: Compilation of movies for the TIRF experiments in Fig. 6 and Supplementary Fig. 9. The white scale bar corresponds to 3 µm and is valid for all movies. TIRF: total internal reflection.

Title: Supplementary Movie 2:
Description: Movies for the TIRF experiments in Fig. 5. The white scale bar corresponds to 3 µm and is valid for all movies. TIRF: total internal reflection
