# Supplementary material for: Conformational coupling of the sialic acid TRAP transporter HiSiaQM with its substrate binding protein HiSiaP
Source: Nat Commun. 2024 Jan 8;15:217. doi: 10.1038/s41467-023-44327-3 (PMC10774421; doi:10.1038/s41467-023-44327-3)
Supplement: Supplementary file 6 — Reporting Summary [file 41467_2023_44327_MOESM6_ESM.pdf]

nature portfolio

Corresponding author(s): Gregor Hagelueken

Last updated by author(s): 04.12.23

Reporting Summary

Nature Portfolio wishes to improve the reproducibility of the work that we publish. This form provides structure for consistency and transparency in reporting. For further information on Nature Portfolio policies, see our Editorial Policies and the Editorial Policy Checklist.

Statistics

For all statistical analyses, confirm that the following items are present in the figure legend, table legend, main text, or Methods section.

n/a	Confirmed	
☐	☒	The exact sample size (*n*) for each experimental group/condition, given as a discrete number and unit of measurement
☐	☒	A statement on whether measurements were taken from distinct samples or whether the same sample was measured repeatedly
☐	☒	The statistical test(s) used AND whether they are one- or two-sided<br>*Only common tests should be described solely by name; describe more complex techniques in the Methods section.*
☒	☐	A description of all covariates tested
☒	☐	A description of any assumptions or corrections, such as tests of normality and adjustment for multiple comparisons
☐	☒	A full description of the statistical parameters including central tendency (e.g. means) or other basic estimates (e.g. regression coefficient) AND variation (e.g. standard deviation) or associated estimates of uncertainty (e.g. confidence intervals)
☐	☒	For null hypothesis testing, the test statistic (e.g. *F*, *t*, *r*) with confidence intervals, effect sizes, degrees of freedom and *P* value noted<br>*Give P values as exact values whenever suitable.*
☒	☐	For Bayesian analysis, information on the choice of priors and Markov chain Monte Carlo settings
☒	☐	For hierarchical and complex designs, identification of the appropriate level for tests and full reporting of outcomes
☒	☐	Estimates of effect sizes (e.g. Cohen's *d*, Pearson's *r*), indicating how they were calculated

Our web collection on statistics for biologists contains articles on many of the points above.

Software and code

Policy information about availability of computer code

Data collection	Software for diffraction data collection at beamline P13 of PETRAIII: mxCube v2
Data analysis	autoproc (v1.1.17), Phenix (v. 1.20), coot (v. 0.9), PyMOL (v.2.3), Molprobity (v 4.5.2), Fiji (v. 1.52p)

For manuscripts utilizing custom algorithms or software that are central to the research but not yet described in published literature, software must be made available to editors and reviewers. We strongly encourage code deposition in a community repository (e.g. GitHub). See the Nature Portfolio guidelines for submitting code & software for further information.

Data

Policy information about availability of data

All manuscripts must include a data availability statement. This statement should provide the following information, where applicable:

- Accession codes, unique identifiers, or web links for publicly available datasets
- A description of any restrictions on data availability
- For clinical datasets or third party data, please ensure that the statement adheres to our policy

The coordinate and diffraction data generated in this study have been deposited in the PDB under accession code 8CP7 [https://doi.org/10.2210/pdb8CP7/pdb]. The movie data generated in this study are provided in the Supplementary Information. Source data are provided with this paper. The coordinate data used in this study are available in the PDB database under accession code 7QE5 [https://doi.org/10.2210/pdb7qe5/pdb] and the Alphafold2 predictions of the HiSiaPQM complexes shown in Fig.1 are available in the Supplementary information of ref. 14 (Peter et al. 2022) [https://doi.org/10.1038/s41467-022-31907-y].

Research involving human participants, their data, or biological material

Policy information about studies with human participants or human data. See also policy information about sex, gender (identity/presentation), and sexual orientation and race, ethnicity and racism.

Reporting on sex and gender	Not applicable for this study.
Reporting on race, ethnicity, or other socially relevant groupings	Not applicable for this study.
Population characteristics	Not applicable for this study.
Recruitment	Not applicable for this study.
Ethics oversight	Not applicable for this study.

Note that full information on the approval of the study protocol must also be provided in the manuscript.

Field-specific reporting

Please select the one below that is the best fit for your research. If you are not sure, read the appropriate sections before making your selection.

☒ Life sciences ☐ Behavioural & social sciences ☐ Ecological, evolutionary & environmental sciences

For a reference copy of the document with all sections, see nature.com/documents/nr-reporting-summary-flat.pdf

Life sciences study design

All studies must disclose on these points even when the disclosure is negative.

Sample size	No sample size calculations were performed. No experiments involving biological specimens were examined such that sample size does not apply. Dozens of crystals were screend to identify the optimal crystal for data collection. The number of crystals screened was random and was not<br>limited by any experimental parameter. Biochemical experiments were confirmed with multiple replicates as detailed in the Methods and Figure Legends.
Data exclusions	No data was excluded.
Replication	The results of the biochemical experiments could be confirmed by multiple successful replications, as detailed in Methods and in the figure legends.
Randomization	No experiment involving animals or humans was performed in this study, therefore randomization is not applicable for this study
Blinding	Blinding is not applicable for this study as no experiment involving humans or animals was performed

Reporting for specific materials, systems and methods

We require information from authors about some types of materials, experimental systems and methods used in many studies. Here, indicate whether each material, system or method listed is relevant to your study. If you are not sure if a list item applies to your research, read the appropriate section before selecting a response.

Materials & experimental systems

n/a	Involved in the study	
☐	☒	Antibodies
☒	☐	Eukaryotic cell lines
☒	☐	Palaeontology and archaeology
☒	☐	Animals and other organisms
☒	☐	Clinical data
☒	☐	Dual use research of concern
☒	☐	Plants

Methods

n/a	Involved in the study	
☒	☐	ChIP-seq
☒	☐	Flow cytometry
☒	☐	MRI-based neuroimaging

Antibodies

Antibodies used	The VHHQM3 protein was produced by immunozation of an alpaca with the HiSiaQM protein, as described in detail in our previous

publication (Peter, M. F. et al. Structural and mechanistic analysis of a tripartite ATP-independent periplasmic TRAP transporter. Nat Commun 13, 4471 (2022)). The VHH is not commercially available.

Validation

The VHH was recombinantely produced in E. coli. And it was extensively tested and characterized in our previous publication (Peter, M. F. et al. Structural and mechanistic analysis of a tripartite ATP-independent periplasmic TRAP transporter. Nat Commun 13, 4471 (2022)). In this manuscript, the same VHH construct was used and behaved as expected in all experiments.

Plants

Seed stocks

Report on the source of all seed stocks or other plant material used. If applicable, state the seed stock centre and catalogue number. If plant specimens were collected from the field, describe the collection location, date and sampling procedures.

Novel plant genotypes

Describe the methods by which all novel plant genotypes were produced. This includes those generated by transgenic approaches, gene editing, chemical/radiation-based mutagenesis and hybridization. For transgenic lines, describe the transformation method, the number of independent lines analyzed and the generation upon which experiments were performed. For gene-edited lines, describe the editor used, the endogenous sequence targeted for editing, the targeting guide RNA sequence (if applicable) and how the editor was applied.

Authentication

Describe any authentication procedures for each seed stock used or novel genotype generated. Describe any experiments used to assess the effect of a mutation and, where applicable, how potential secondary effects (e.g. second site T-DNA insertions, mosiacism, off-target gene editing) were examined.
